# Supplementary material for: Modeling Protein–Glycan Interactions with HADDOCK
Source: J Chem Inf Model. 2024 Oct 3;64(19):7816–25. doi: 10.1021/acs.jcim.4c01372 (PMC11480977; doi:10.1021/acs.jcim.4c01372)
Supplement: Supplementary file 2 — ci4c01372_si_002.pdf [file ci4c01372_si_002.pdf]

Supplementary Information

Modelling Protein-Glycan Interactions with HADDOCK

Anna Ranaudo[1,2], Marco Giulini[2], Angela Pelissou Ayuso[2], Alexandre M. J. J. Bonvin[2]*

[1] Department of Earth and Environmental Sciences, University of Milano-Bicocca, Piazza della Scienza 1, 20126 Milan, Italy

[2] Bijvoet Centre for Biomolecular Research, Faculty of Science - Chemistry, Utrecht University, Padualaan 8, 3584 Utrecht, CH, The Netherlands

[*]a.m.j.j.bonvin@uu.nl

Table of Contents

- Text S1. Details of the preparation of proteins and glycans structures……..………..…p. S2
- Text S2. Description of HADDOCK3 modules used in this work…………………….p. S2
- Text S3. Details of the glycans conformational sampling protocol…………..…………...p. S5
- Table S1. SNFG representation of the glycans……………………………………..p. S6
- Table S2. Modules and parameters used for bound docking…………………………p. S17
- Table S3. Modules and parameters used for unbound docking………………………p. S18
- Table S4. Glycans conformational sampling scenarios………………………………p. S19
- Figure S1. Example of HADDOCK models satisfying the quality thresholds……......p. S20
- Figure S2. HADDOCK3 performance on the bound dataset………...………………. p. S21
- Figure S3. Glycans RMSD to their bound conformations…………………………... p. S22
- Figure S4. Impact of mdref on glycans conformations……………………………… p. S23
- Figure S5. Impact of the clustering on glycans lowest RMSD………………………. p. S24
- Figure S6. Examples of glycans ensembles of conformations………………………. p. S25
- Figure S7. HADDOCK3 performance with the ensembles of glycans………………p. S26
- Figure S8. Torsion angle analysis of glycosidic linkages: a comparison to HADDOCK flexible refinement models………………………………………………………….. p. S27
- Figure S9. Torsion angle analysis of glycosidic linkages: a comparison to HADDOCK short molecular dynamics refinement models……………………………………….p. S32
- Supplementary References…………………………...……………………..…….p. S37

Text S1: Details of the preparation of proteins and glycans structures

Glycans unbound conformations were generated with the GLYCAM-Web webserver[1,2] with the GLYCAM nomenclature for the carbohydrates residues modified to match the naming recognized by HADDOCK. Prior to docking calculations, all structures were pre-processed with a combination of pdb-tools[3], in-house scripts and manual curation. Heteroatoms such as water molecules, cofactors and ions were removed when not part of the protein – glycan interface. The residues were renumbered to start from 1, with `pdb_reres`, and the chains ID were modified using `pdb_chain` to chain A and chain B for the receptor and the ligand, respectively. In some cases where the receptor consists of more than one chain, these were merged into a single chain, and the residue numbering was shifted if needed to avoid overlap in numbering. Alternative occupancies were removed by selecting the conformation with the highest occupancy value.

Text S2: Description of HADDOCK3 modules used in this work

HADDOCK3 modules used in this work are briefly explained. For further information, please refer to HADDOCK documentation (https://www.bonvinlab.org/haddock3/).

Topology module

[topoaa] This module generates CNS[4] compatible parameters (.param) and topologies (.psf) for each of the input structures. It detects missing atoms, including hydrogens, re-builds them when missing, and builds and writes out topologies (.psf) and coordinates (.pdb) files.
Also refer to:
https://www.bonvinlab.org/haddock3/modules/topology/haddock.modules.topology.topoaa.html

Sampling module

[rigidbody] In this rigid body sampling module the two partners are first separated in space and their orientation randomized, followed by docking using a rigid-body energy minimization protocol. The input molecules are thus treated as rigid bodies. At this stage, Ambiguous Interaction Restraints (AIRs) are included to drive the docking process.
Default parameters can be found at:

https://www.bonvinlab.org/haddock3/modules/sampling/haddock.modules.sampling.rigidbody.html

Refinement modules

[flexref] This module performs a semi-flexible refinement by simulated annealing in torsion angle space during which the interfaces are considered as flexible. The simulated annealing protocol consists of four stages: i) high temperature rigid body molecular dynamics; ii) rigid body simulated annealing; iii) semi-flexible simulated annealing with flexible side-chains at the interface; and iv) semi-flexible simulated annealing with fully flexible interface (both backbone and side-chains). The number of steps and the temperature of the different stages can be adjusted. Ambiguous Interaction Restraints (AIRs) are included. Details of this module can be found at: https://www.bonvinlab.org/haddock3/modules/refinement/haddock.modules.refinement.flexref.html

[mdref] This module performs a short molecular dynamics simulation in explicit solvent (a 8Å layer of water molecules is generated around the molecules. In this work, this module was used to generate an ensemble of glycans conformations.

The MD refinement consists of four sequential stages, each with a pre-defined number of steps: i) short energy minimization (`nemsteps`); ii) three stages of molecular dynamics to reach 300K (at 100, 200 and 300K) (`waterheatsteps`); iii) molecular dynamics at 300K (`watersteps`); iv) three stages of molecular dynamics, to reach 100K (at 300, 200 and 100K) (`watercoolsteps`). The number of models generated is defined by the parameter sampling_factor. Refer to the following link for the other parameters: https://www.bonvinlab.org/haddock3/modules/refinement/haddock.modules.refinement.mdref.html

Analysis modules

[rmsdmatrix] This module calculates the RMSD matrix between all models generated in the previous step (e.g., after rigidbody or flexref). The residues considered for the alignment and RMSD calculation are provided with the expandable parameter `resdic_`.

Please refer to the following link for a full explanation of the module and the parameters: https://www.bonvinlab.org/haddock3/modules/analysis/haddock.modules.analysis.rmsdmatrix.html.

[clustrmsd] This module takes as input the RMSD matrix calculated in the previous step and performs a hierarchical clustering procedure, leveraging scipy routines for this purpose. Briefly,

the models are grouped into a progressively coarser hierarchy of clusters (i.e., the dendrogram). The main parameters are:

- `criterion`, which defines the prescription to cut the dendrogram and obtain the desired clusters. In this work, depending on the stage of the protocol, we used both `criterion = maxclust` and `criterion = distance`
- `linkage`, which governs the way clusters are merged together in the creation of the dendrogram. We used the default `linkage = average`
- `min_population`: it is the minimum number of models that should be present in a cluster to consider it. This applies only for `criterion = distance`; if `criterion = maxclust`, the value is ignored.
- `clust_cutoff`: value of distance that separates distinct clusters (only for `criterion = distance`)
- `n_clusters`: number of desired clusters (only for `criterion = maxclust`)

For further information, refer to the link:
https://www.bonvinlab.org/haddock3/modules/analysis/haddock.modules.analysis.clustrmsd.html

[seletopclusts] This module selects a number of models from a number of clusters. The selection is based on the score of the models within the clusters. In our protocol, we retained the top 5 rigidbody models from all the 50 or 150 clusters produced at the previous step.
Also refer to the following link:
https://www.bonvinlab.org/haddock3/modules/analysis/haddock.modules.analysis.seletopclusts.html

[caprieval] This module calculates the CAPRI metrics[5] for the given input models. If a reference structure is provided all metrics are calculated with respect to that structure. If none are provided the best scoring model is used as reference. By default, the following metrics are calculated: FNAT (fraction of native contacts); IRMSD (interface root mean square deviation); LRMSD (ligand root mean square deviation); DOCKQ[6], a measure of the quality of the docked model based on FNAT, IRMSD and LRMSD; ILRMSD (interface ligand root mean square deviation).
In this work, for the reasons explained in the main text, the ILRMSD was used to evaluate the quality of both single models (capri_ss.tsv: file) and clustered models (capri_clt.tsv).
The default parameters are shown at the following link:
https://www.bonvinlab.org/haddock3/modules/analysis/haddock.modules.analysis.caprieval.html

Text S3: Details of the glycans conformational sampling protocol

Glycans conformations generated from the GLYCAM-Web webserver were used as starting points for conformational sampling. For 11 out of 55 glycans two conformations were generated by the webserver (four for 1OH4); for those cases, all conformations were used. Conformational sampling and analysis were performed with HADDOCK3 using the following modules: `topoaa`, `mdref`, `rmsdmatrix`, `clustrmsd`. After the creation of the topology (module `topoaa`), conformational sampling in water was performed with the water refinement module (`mdref`), defining the glycans as fully flexible (parameters: `nfle1 = 1; fle_sta_1_1 = 1; fle_end_1_1 = 7`). At this stage, different scenarios were tested in terms of number of steps and number of models, as specified in Table S4. Three scenarios were run on 100 models with increasing number of steps / simulation time (sf100-x1, sf100-x8, sf100-x16). Then, the number of models was increased to 400 while the simulation time was the same of two of the scenarios previously listed (sf400-x1 and sf400-x16). The overall time of the simulations ranges from 185000 (sf100-x1) to 11240000 steps (sf400-x16).
For assessing the conformational variability over the sampled trajectory, all-atom RMSD were calculated for all the generated conformations, with respect to the bound conformations, with the HADDOCK3 `rmsdmatrix` module. RMSD distributions of the conformations obtained with the sampling procedures were plotted together with the RMSD calculated for the webserver-generated conformations with respect to the bound conformations.

The RMSD matrix between all the conformations generated was calculated with the `rmsdmatrix` module, by specifying, through the parameter '`resdic_`', the residues to be considered for the alignment and the RMSD calculation. The `clustrmsd` module was then exploited for clustering the conformations, with the following parameters: `criterion = maxclust, linkage = average, n_clusters = 10 (or 20)`. The '`maxclust`' criterion clusters the structure in such a way to give a fixed number of clusters, defined by the parameter `n_clusters`. The linkage governs the way clusters are merged in the creation of the dendrogram, i.e., it defines the method for calculating the distance between the newly formed cluster and each object which does not belong to a cluster yet. RMSD with respect to the bound conformations were calculated for the clusters centers, i.e. the points having the lower distance to all the other points in the cluster. RMSD of to the cluster centers were plotted together with the overall sampling distribution to assess whether the clustering can capture the models that are closer to the glycan experimental structure. The centers of the clusters were then used as an ensemble for further docking calculations.

Table S1. SNFG representation of the glycans.

For each entry in the bound dataset, identified by its Protein Data Bank (PDB) ID, the glycan sequence, its group, and the Symbol Nomenclature For Glycans (SNFG)[7,8] are provided. The SNFG figures, originally generated with GlycanBuilder[9] were downloaded from the Protein Data Bank[10].

PDB_ID (bound complex)	Sequence of the glycan	Glycan group	SNFG representation
3OAU	a-D-Manp-(1->2)-a-D-Manp	SL	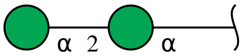
1WU6	b-D-Xylp-(1->4)-b-D-Xylp	SL	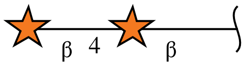
3N17	b-D-GlcpNAc-(1->4)-b-D-GlcpNAc	SL	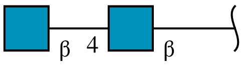
1W6P	b-D-Galp-(1->4)-a-D-GlcpNAc	SL	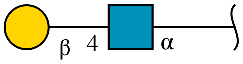
2IT6	a-D-Manp-(1->2)-a-D-Manp	SL	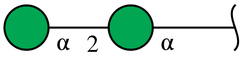
3VV1	b-D-Galp-(1->4)-a-L-Fucp	SL	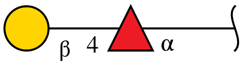
4R9F	b-D-Manp-(1->4)-b-D-Manp	SL	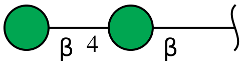
5T4Z	a-D-Manp-(1->2)-a-D-Manp-(1->3)-a-D-Manp	SL	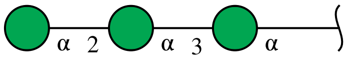

2XOM	b-D-Galp-(1->4)-b-D-Galp-(1->4)-b-D-Galp	SL	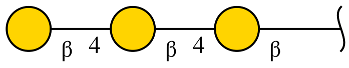
4QPW	b-D-Xylp-(1->4)-b-D-Xylp-(1->4)-b-D-Xylp	SL	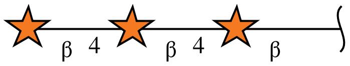
4D5I	b-D-Xylp-(1->4)-b-D-Xylp-(1->4)-b-D-Xylp	SL	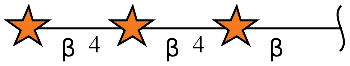
2G7C	a-D-Galp-(1->3)-b-D-Galp-(1->4)-b-D-GlcpNAc	SL	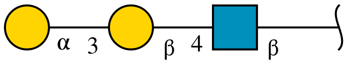
2YP3	a-NeupAc-(2->6)-b-D-Galp-(1->4)-b-D-GlcpNAc	SL	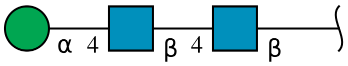
5HZB	a-L-Fucp-(1->2)-b-D-Galp-(1->4)-a-D-Glcp	SL	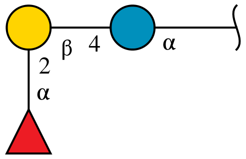
1UZ8	a-L-Fucp-(1->3)-[b-D-Galp-(1->4)]-b-D-GlcpNAc	SB	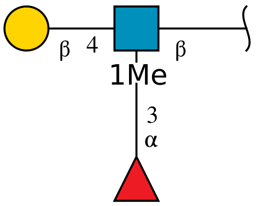
1JPC	a-D-Manp-(1->3)-[a-D-Manp-(1->6)]-a-D-Manp	SB	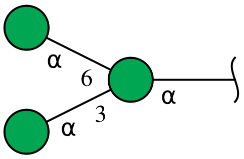

2WRA	a-D-Manp-(1->3)-[a-D-Manp-(1->6)]-a-D-Manp	SB	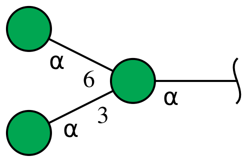
5HZA	a-L-Fucp-(1->3)-[b-D-Galp-(1->4)]-b-D-Glcp	SB	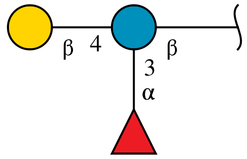
5V6F	a-D-Manp-(1->3)-[a-D-Manp-(1->6)]-b-D-Manp	SB	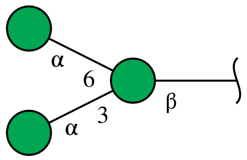
6R3M	b-D-Glcp-(1->4)-b-D-Glcp-(1->4)-b-D-Glcp-(1->3)-b-D-Glcp	LL	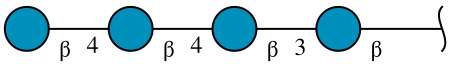
1JDC	a-D-Glcp-(1->4)-a-D-Glcp-(1->4)-a-D-Glcp-(1->4)-a-D-Glcp	LL	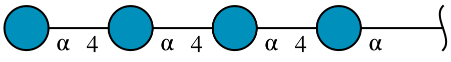
3WH1	b-D-GlcpNAc-(1->4)-b-D-GlcpNAc-(1->4)-b-D-GlcpNAc-(1->4)-b-D-GlcpNAc	LL	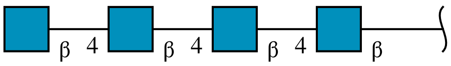
4YG0	b-D-Galp-(1->4)-b-D-GlcpNAc-(1->3)-b-D-Galp-(1->4)-b-D-Glcp	LL	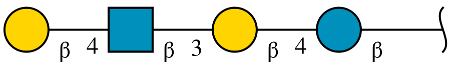
1S3K	a-L-Fucp-(1->3)-[a-L-Fucp-(1->2)-b-D-Galp-(1->4)]-a-D-GlcpNAc	LB	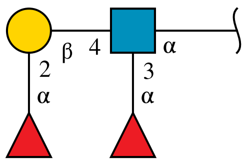

1SL5	a-L-Fucp-(1->3)-[b-D-Galp-(1->4)]-b-D-GlcpNAc-(1->3)-b-D-Galp	LB	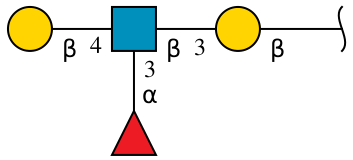
2I74	a-D-Manp-(1->3)-[a-D-Manp-(1->6)]-a-D-Manp-(1->6)-a-D-Manp	LB	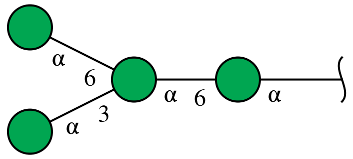
2CHB	a-NeupAc-(2->3)-[b-D-Galp-(1->3)-b-D-GalpNAc-(1->4)]-b-D-Galp	LB	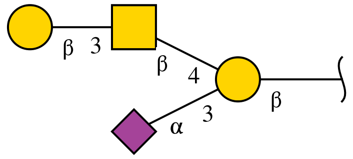
6BE4	b-D-GlcpNAc-(1->6)-b-D-GlcpNAc-(1->6)-b-D-GlcpNAc-(1->6)-b-D-GlcpNAc-(1->6)-b-D-GlcpNAc	LL	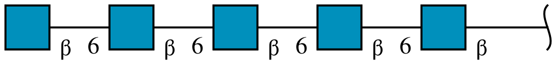
1W8U	b-D-Manp-(1->4)-b-D-Manp-(1->4)-b-D-Manp-(1->4)-b-D-Manp-(1->4)-b-D-Manp	LL	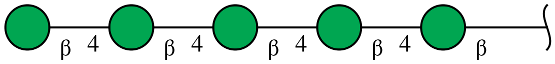
2WAB	b-D-Glcp-(1->4)-b-D-Glcp-(1->4)-b-D-Glcp-(1->4)-b-D-Glcp-(1->4)-b-D-Glcp	LL	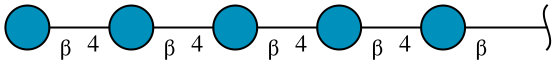
2YP4	a-NeupAc-(2->6)-b-D-Galp-(1->4)-b-D-GlcpNAc-(1->3)-b-D-Galp-(1->4)-b-D-Glcp	LL	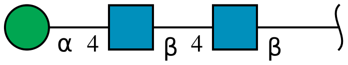
1GUI	b-D-Glcp-(1->3)-b-D-Glcp-(1->3)-b-D-Glcp-(1->3)-b-D-Glcp-(1->3)-b-D-Glcp-(1->3)-b-D-Glcp	LL	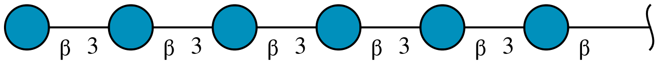

1GWL	b-D-Manp-(1->4)-b-D-Manp-(1->4)-b-D-Manp-(1->4)-b-D-Manp-(1->4)-b-D-Manp-(1->4)-b-D-Manp	LL	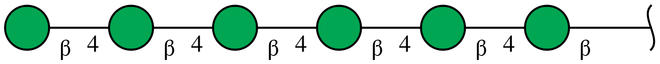
1GWM	b-D-Glcp-(1->4)-b-D-Glcp-(1->4)-b-D-Glcp-(1->4)-b-D-Glcp-(1->4)-b-D-Glcp-(1->4)-a-D-Glcp	LL	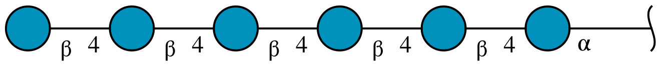
6N35	a-D-Manp-(1->2)-a-D-Manp	SL	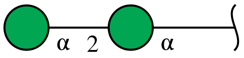
1C1L	b-D-Galp-(1->4)-b-D-Glcp	SL	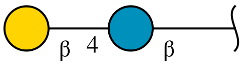
1I3H	a-D-Manp-(1->2)-a-D-Manp	SL	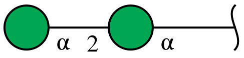
1KJL	b-D-Galp-(1->4)-b-D-GlcpNAc	SL	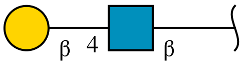
1PWB	a-D-Glcp-(1->4)-a-D-Glcp	SL	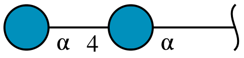
1SLT	b-D-Galp-(1->4)-a-D-GlcpNAc	SL	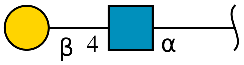
2RDK	a-D-Manp-(1->2)-a-D-Manp	SL	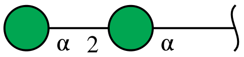
2ZKN	b-D-Galp-(1->4)-b-D-Glcp	SL	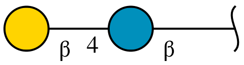

3G83	a-D-Manp-(1->2)-a-D-Manp	SL	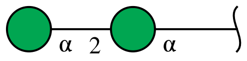
3P5H	b-D-Glcp-(1->3)-b-D-Glcp	SL	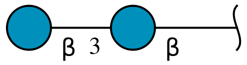
5GAL	b-D-Galp-(1->4)-b-D-GlcpNAc	SL	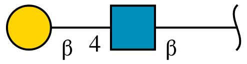
5YRG	a-D-Glcp-(1->6)-b-D-Glcp	SL	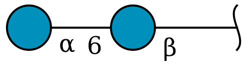
6H9Y	b-D-Galp-(1->3)-b-D-GlcpNAc	SL	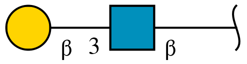
2J1V	a-L-Fucp-(1->2)-b-D-Galp-(1->4)-b-D-GlcpNAc	SL	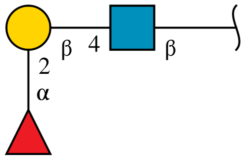
2Y6G	b-D-Glcp-(1->4)-b-D-Glcp-(1->4)-b-D-Glcp	SL	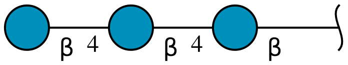
154L	b-D-GlcpNAc-(1->4)-b-D-GlcpNAc-(1->4)-b-D-GlcpNAc	SL	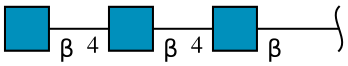
3AOF	b-D-Manp-(1->4)-b-D-Manp-(1->4)-b-D-Manp	SL	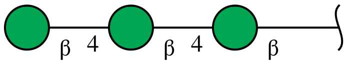
5AWQ	a-D-Glcp-(1->6)-a-D-Glcp-(1->4)-a-D-Glcp	SL	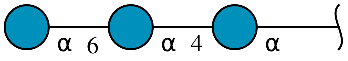

5JU9	b-D-Manp-(1->4)-b-D-Manp-(1->4)-a-D-Manp	SL	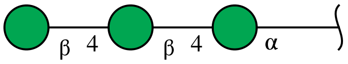
1QFO	a-NeupAc-(2->3)-b-D-Galp-(1->4)-a-D-Glcp	SL	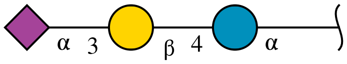
2VXJ	a-D-Galp-(1->3)-b-D-Galp-(1->4)-b-D-Glcp	SL	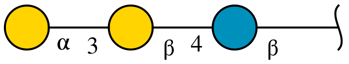
3NV4	a-NeupAc-(2->3)-b-D-Galp-(1->4)-b-D-Glcp	SL	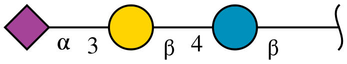
4MBY	a-NeupAc-(2->3)-b-D-Galp-(1->4)-b-D-Glcp	SL	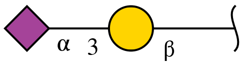
6HA0	a-L-Fucp-(1->2)-b-D-Galp-(1->3)-b-D-GlcpNAc	SL	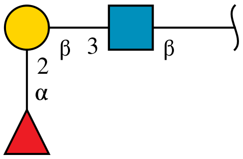
3P5G	a-L-Fucp-(1->2)-[a-D-Galp-(1->3)]-b-D-Galp	SB	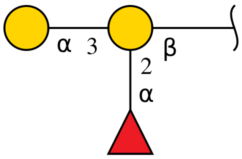
6MSY	a-D-Manp-(1->2)-a-D-Manp-(1->2)-a-D-Manp-(1->3)-a-D-Manp	LL	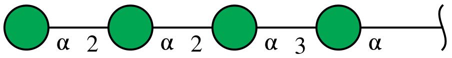
2J72	a-D-Glcp-(1->4)-a-D-Glcp-(1->4)-a-D-Glcp-(1->4)-a-D-Glcp	LL	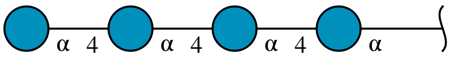
2J73	a-D-Glcp-(1->6)-a-D-Glcp-(1->4)-a-D-Glcp-(1->4)-a-D-Glcp	LL	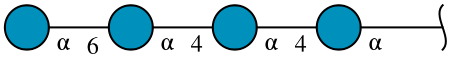

3ACH	b-D-Glcp-(1->4)-b-D-Glcp-(1->4)-b-D-Glcp-(1->4)-b-D-Glcp	LL	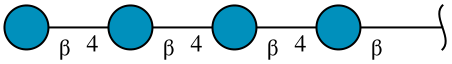
4XUR	b-D-Xylp-(1->4)-b-D-Xylp-(1->4)-b-D-Xylp-(1->4)-b-D-Xylp	LL	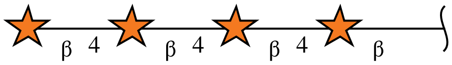
1KQZ	b-D-GlcpNAc-(1->4)-b-D-GlcpNAc-(1->4)-b-D-GlcpNAc-(1->4)-b-D-GlcpNAc	LL	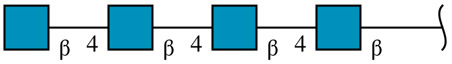
1LMQ	b-D-GlcpNAc-(1->4)-b-D-GlcpNAc-(1->4)-b-D-GlcpNAc-(1->4)-a-D-GlcpNAc	LL	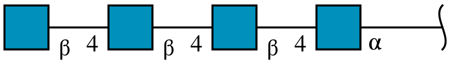
1UU6	b-D-Glcp-(1->4)-b-D-Glcp-(1->4)-b-D-Glcp-(1->4)-b-D-Glcp	LL	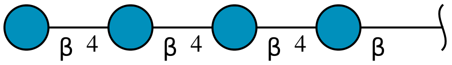
2BOF	b-D-Glcp-(1->4)-b-D-Glcp-(1->4)-b-D-Glcp-(1->4)-b-D-Glcp	LL	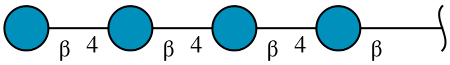
4DQJ	b-D-GlcpNAc-(1->4)-b-D-GlcpNAc-(1->4)-b-D-GlcpNAc-(1->4)-b-D-GlcpNAc	LL	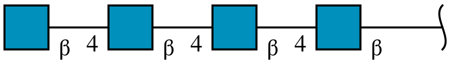
5GY0	b-D-Glcp-(1->4)-b-D-Glcp-(1->4)-b-D-Glcp-(1->4)-b-D-Glcp	LL	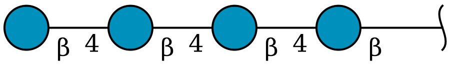
4YFZ	b-D-Galp-(1->3)-b-D-GlcpNAc-(1->3)-b-D-Galp-(1->4)-b-D-Glcp	LL	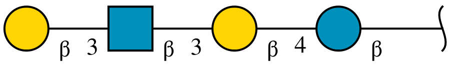
2J1T	a-L-Fucp-(1->3)-[a-L-Fucp-(1->2)-b-D-Galp-(1->4)]-b-D-GlcpNAc	LB	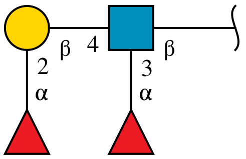

2XJR	a-D-Manp-(1->2)-a-D-Manp-(1->3)-[a-D-Manp-(1->6)]-a-D-Manp	LB	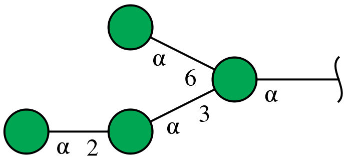
3ZWE	a-L-Fucp-(1->2)-[a-D-Galp-(1->3)]-b-D-Galp-(1->4)-b-D-Glcp	LB	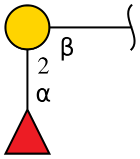
2Z8L	a-L-Fucp-(1->3)-[a-NeupAc-(2->3)-b-D-Galp-(1->4)]-b-D-GlcpNAc	LB	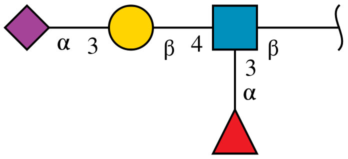
1GNY	b-D-Xylp-(1->4)-b-D-Xylp-(1->4)-b-D-Xylp-(1->4)-b-D-Xylp-(1->4)-b-D-Xylp	LL	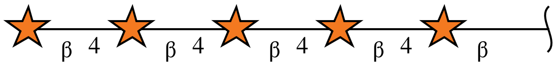
1OF4	b-D-Manp-(1->4)-b-D-Manp-(1->4)-b-D-Manp-(1->4)-b-D-Manp-(1->4)-b-D-Manp	LL	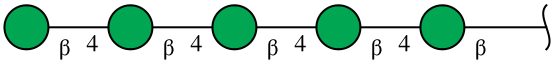
1UXX	b-D-Xylp-(1->4)-b-D-Xylp-(1->4)-b-D-Xylp-(1->4)-b-D-Xylp-(1->4)-b-D-Xylp	LL	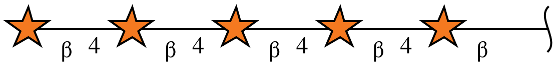
2ZEX	b-D-Glcp-(1->4)-b-D-Glcp-(1->4)-b-D-Glcp-(1->4)-b-D-Glcp-(1->4)-b-D-Glcp	LL	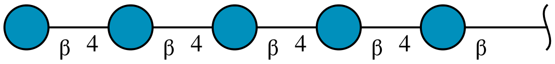
3OEB	b-D-Manp-(1->4)-b-D-Manp-(1->4)-b-D-Manp-(1->4)-b-D-Manp-(1->4)-a-D-Manp	LL	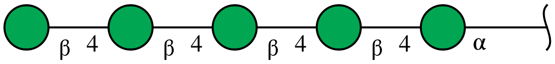
1KQY	b-D-GlcpNAc-(1->4)-b-D-GlcpNAc-(1->4)-b-D-GlcpNAc-(1->4)-	LL	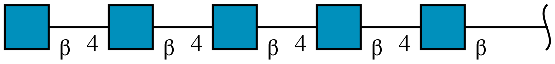

	b-D-GlcpNAc-(1->4)-b-D-GlcpNAc		
5VX5	a-L-Fucp-(1->2)-b-D-Galp-(1->3)-b-D-GlcpNAc-(1->3)-b-D-Galp-(1->4)-b-D-Glcp	LL	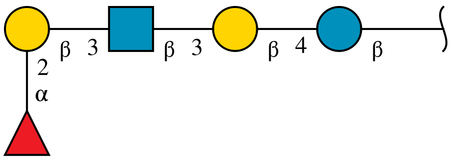
5VX9	a-L-Fucp-(1->2)-b-D-Galp-(1->3)-b-D-GlcpNAc-(1->3)-b-D-Galp-(1->4)-b-D-Glcp	LL	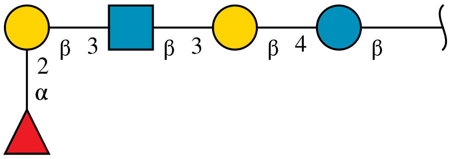
3AP9	a-L-Fucp-(1->3)-[b-D-Galp-(1->4)]-b-D-GlcpNAc-(1->3)-b-D-Galp-(1->4)-b-D-Glcp	LB	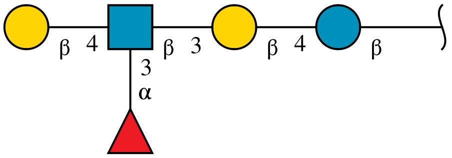
6UG7	a-NeupAc-(2->3)-b-D-Galp-(1->3)-b-D-GalpNAc-(1->3)-a-D-Galp-(1->4)-b-D-Galp-(1->4)-b-D-Glcp	LL	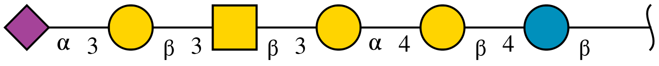
1PMH	b-D-Manp-(1->4)-b-D-Manp-(1->4)-b-D-Manp-(1->4)-b-D-Manp-(1->4)-b-D-Manp-(1->4)-a-D-Manp	LL	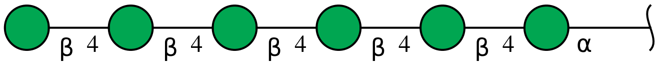
4HK8	b-D-Xylp-(1->4)-b-D-Xylp-(1->4)-b-D-Xylp-(1->4)-b-D-Xylp-(1->4)-b-D-Xylp-(1->4)-b-D-Xylp	LL	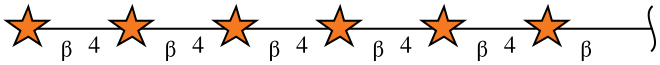
1OH4	b-D-Manp-(1->4)-[a-D-Galp-(1->6)]-b-D-Manp-(1->4)-[a-D-Galp-(1->6)]-b-D-Manp-(1->4)-b-D-Manp-(1->4)-b-D-Manp	LB	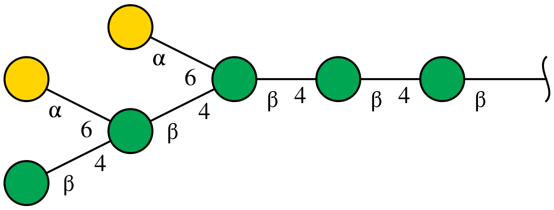

2VUZ	b-D-GlcpNAc-(1->2)-a-D-Manp-(1->3)-[b-D-GlcpNAc-(1->2)-a-D-Manp-(1->6)]-b-D-Manp-(1->4)-b-D-GlcpNAc-(1->4)-b-D-GlcpNAc	LB	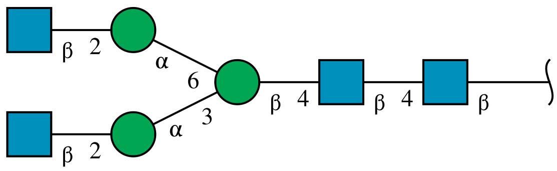

Table S2. Modules and parameters used for *bound docking*.

Protocol bound dataset		
**Stage**	**Module**	**Parameters**
1	topoaa	
2	rigidbody	sampling = 1000<br>w_vdw= 0.01 (default), 1.0 (vdW)<br>ambig_fname = /path/to/tbl/file
3	caprieval	reference_fname = /path/to/reference/pdb
4	rmsdmatrix	resdic_A = [interface residues of the protein]<br>resdic_B = [interface (**ti-aa**) or all (**tip-ap**) residues of the glycan]
5	clustrmsd	criterion = distance<br>linkage = average<br>min_population = 4<br>clust_cutoff = 2.5 Å
6	caprieval	reference_fname = /path/to/reference/pdb

Table S3. Modules and parameters used for *unbound docking*.

**Protocol unbound dataset**		
**Stage**	**Module**	**Parameters**
1	topoaa	
2	rigidbody	sampling = 1000, 4000 (ensemble)<br>w_vdw= 1.0 (vdW)<br>ambig_fname = /path/to/tbl/file
3	caprieval	reference_fname = /path/to/reference/pdb
4	rmsdmatrix	resdic_A = [interface residues of the protein]<br>resdic_B = [all residues of the glycan]
5	clustrmsd	criterion = maxclust<br>n_clusters = 50, 150 (ensemble)
6	seletopclusts	top_models = 5
7	caprieval	reference_fname = /path/to/reference/pdb
8	flexref	tolerance = 5<br>nemsteps = 200<br>mdsteps_rigid = 500<br>mdsteps_cool1 = 500<br>mdsteps_cool2 = 1000<br>mdsteps_cool3 = 1000<br>ambig_fname = /path/to/tbl/file
9	caprieval	reference_fname = /path/to/reference/pdb
10	rmsdmatrix	resdic_A = [interface residues of the protein]<br>resdic_B = [all residues of the glycan]
11	clustrmsd	criterion = distance<br>linkage = average<br>min_population = 4<br>clust_cutoff = 2.5 Å
12	caprieval	reference_fname = /path/to/reference/pdb

Table S4. Glycans conformational sampling scenarios.

scenario_name	Sampling_factor	waterheatsteps	watersteps	watercoolsteps	Total number of steps
Default mdref values	1	100	1250	500	
sf100-x1	100	100	1250	500	185000
sf100-x8	100	100	10000	4000	1410000
sf100-x16	100	100	20000	8000	2810000
sf400-x1	400	100	1250	500	740000
sf400-x16	400	100	20000	8000	11240000

Changes with respect to the scenario sf100-x1 are highlighted in blue. Scenario sf100-x1 follows default parameter setting except for the sampling factor (i.e. number of models generated). The last column reports the total number of steps defined as: sampling_factor * (waterheatsteps + watersteps + watercoolsteps).

Figure S1

Example of HADDOCK models for three different PDB files satisfying the four different quality thresholds highlighted in the main text, namely high, medium, acceptable and near acceptable. Short glycans are clearly more sensitive to small deviations, thus making the near acceptable (IL-RMSD between 3.0 Å and 4.0 Å) threshold too lenient. For long linear glycans (2ZEX in the figure), near acceptable models can still be useful for downstream analysis.

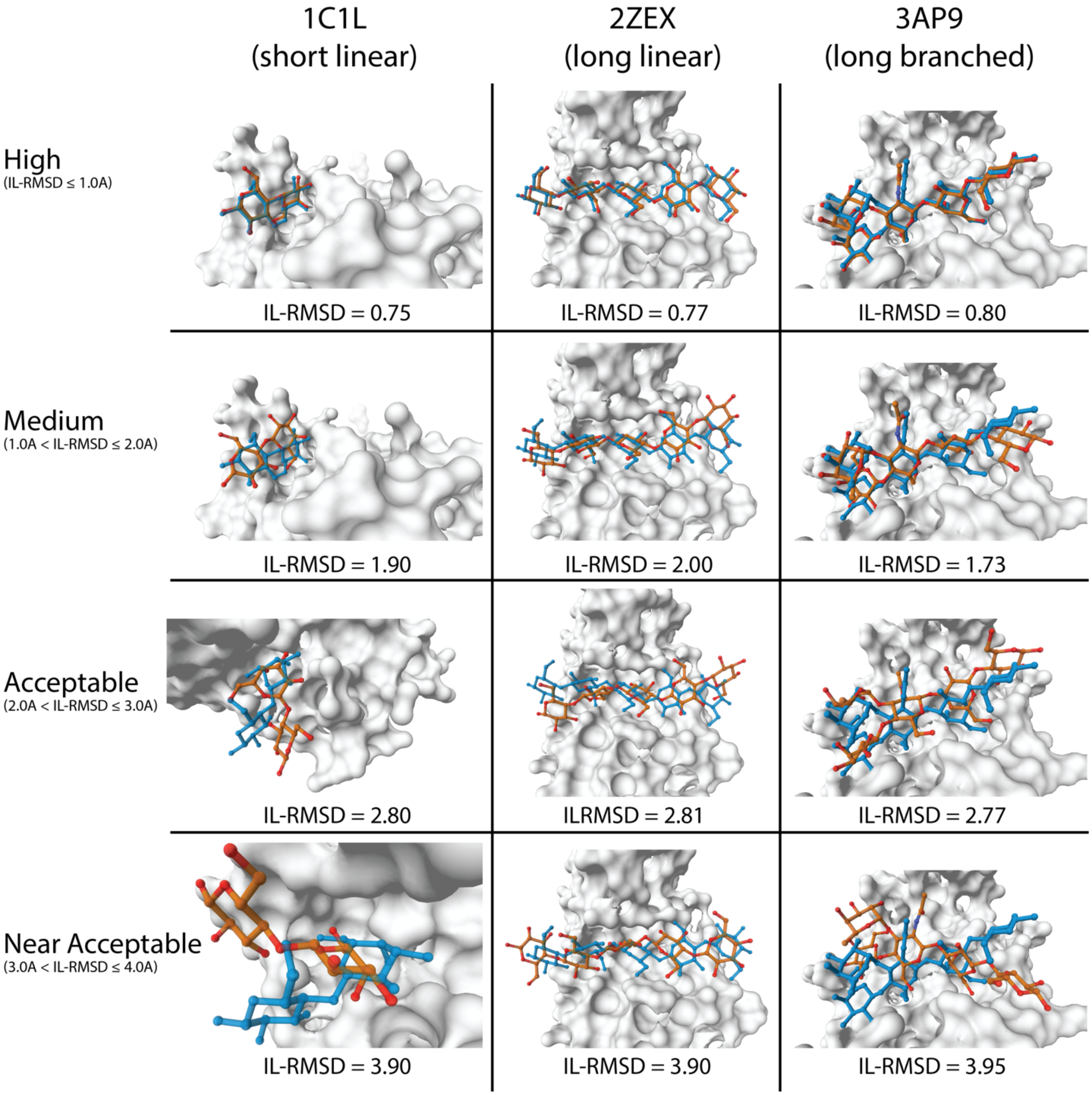

Figure S2

HADDOCK3 performance on the *bound dataset*, as a function of glycans size (**S**: glycans composed by three or less monosaccharide units, or **L** by more than three) and connectivity (**L**: linear, or **B**: branched). The left column shows the performance using both the protein and glycan interface residues as active (**ti-aa**) and the right column using the protein interface residues as active and the entire glycan as passive (**tip-ap**). Success rates are calculated for the top (T) 1, 5, 10, 50, 100, and 200 models.

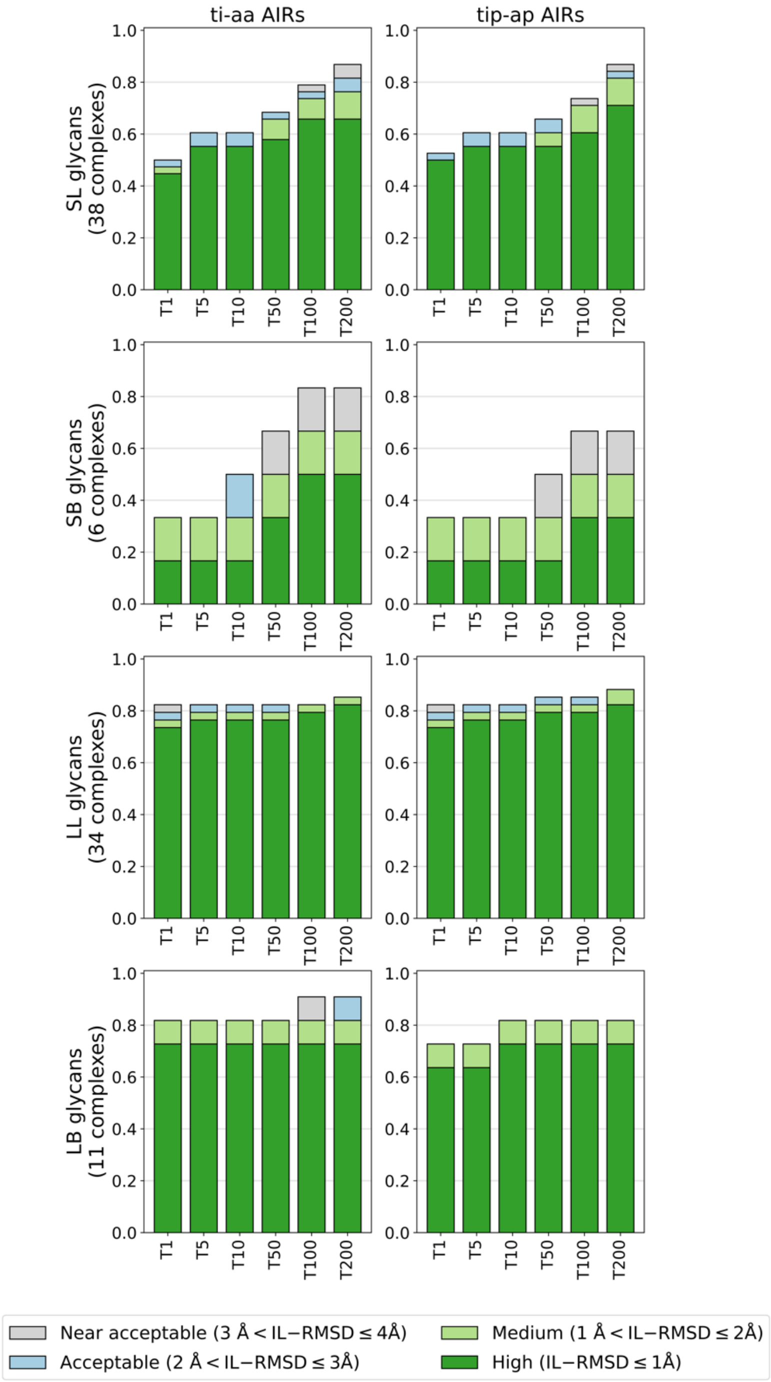

Figure S3

Violin plot showing the distribution of glycan RMSD between the conformations generated with the GLYCAM server and the corresponding bound structure for the three categories of glycans **SL-SB**, **LL**, and **LB**. Mean, maximum and minimum values are indicated in the plot.

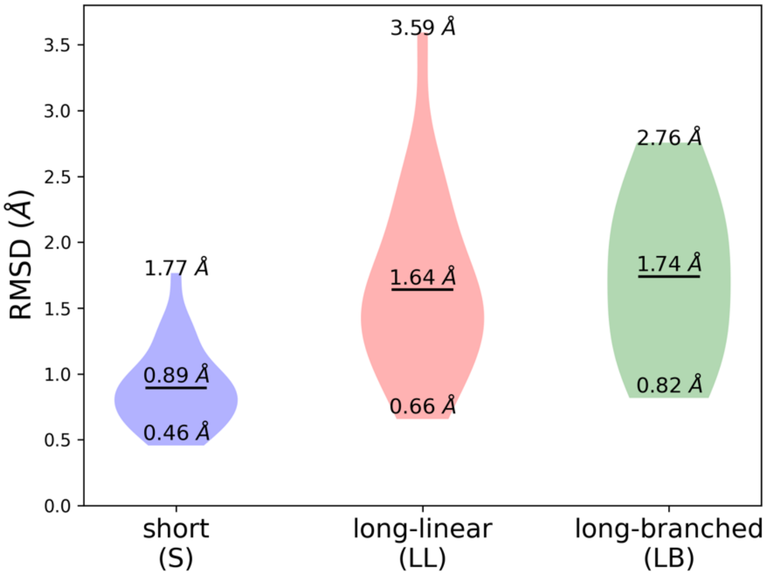

Figure S4

Boxplots of the reference glycan RMSD distribution of the GLYCAM server-generated conformations (left) and the lowest RMSD to the bound form obtained with the 5 sampling scenarios. The comparison is shown for the three groups of glycans: **SL-SB** (top), **LL** (middle), and **LB** (bottom).

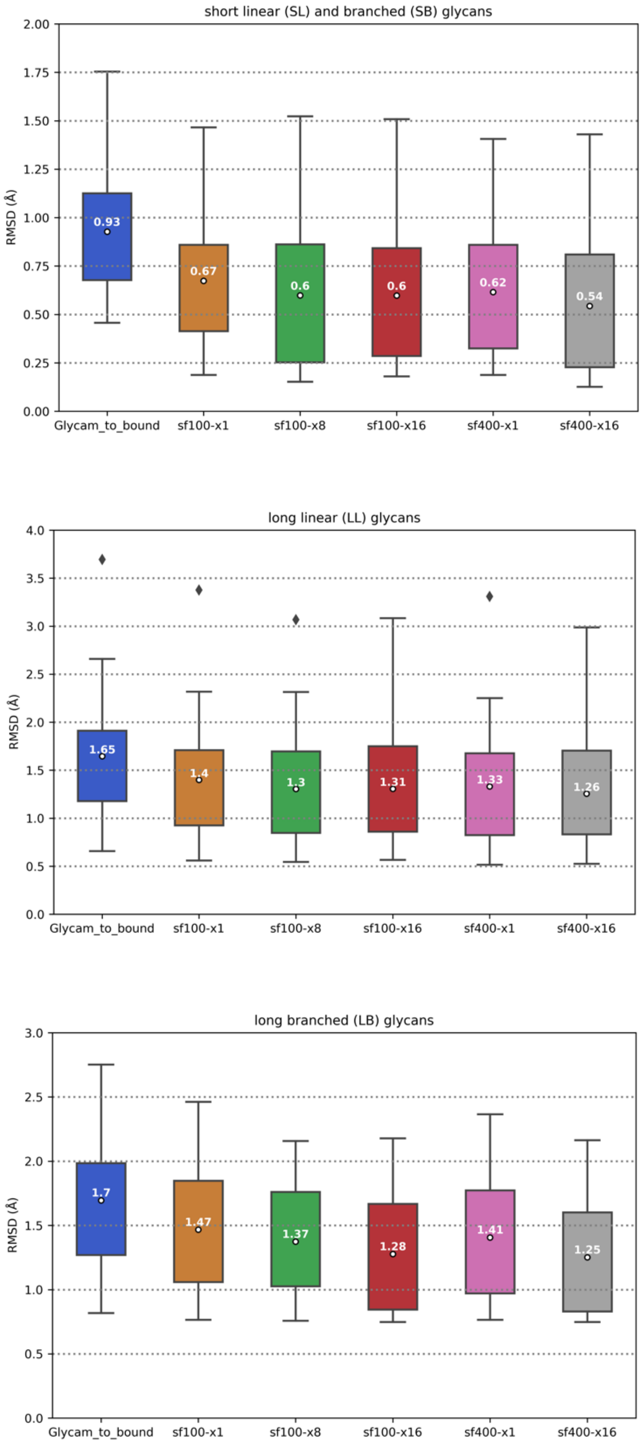

Figure S5

Comparison between lowest RMSDs (glycans sampled conformations with respect to the bound forms) after clustering vs lowest RMSDs from the overall sampling. The comparison is shown for the three groups of glycans: short (**SL-SB**, top left), long linear (**LL**, top right), and long branched (**LB**, bottom).

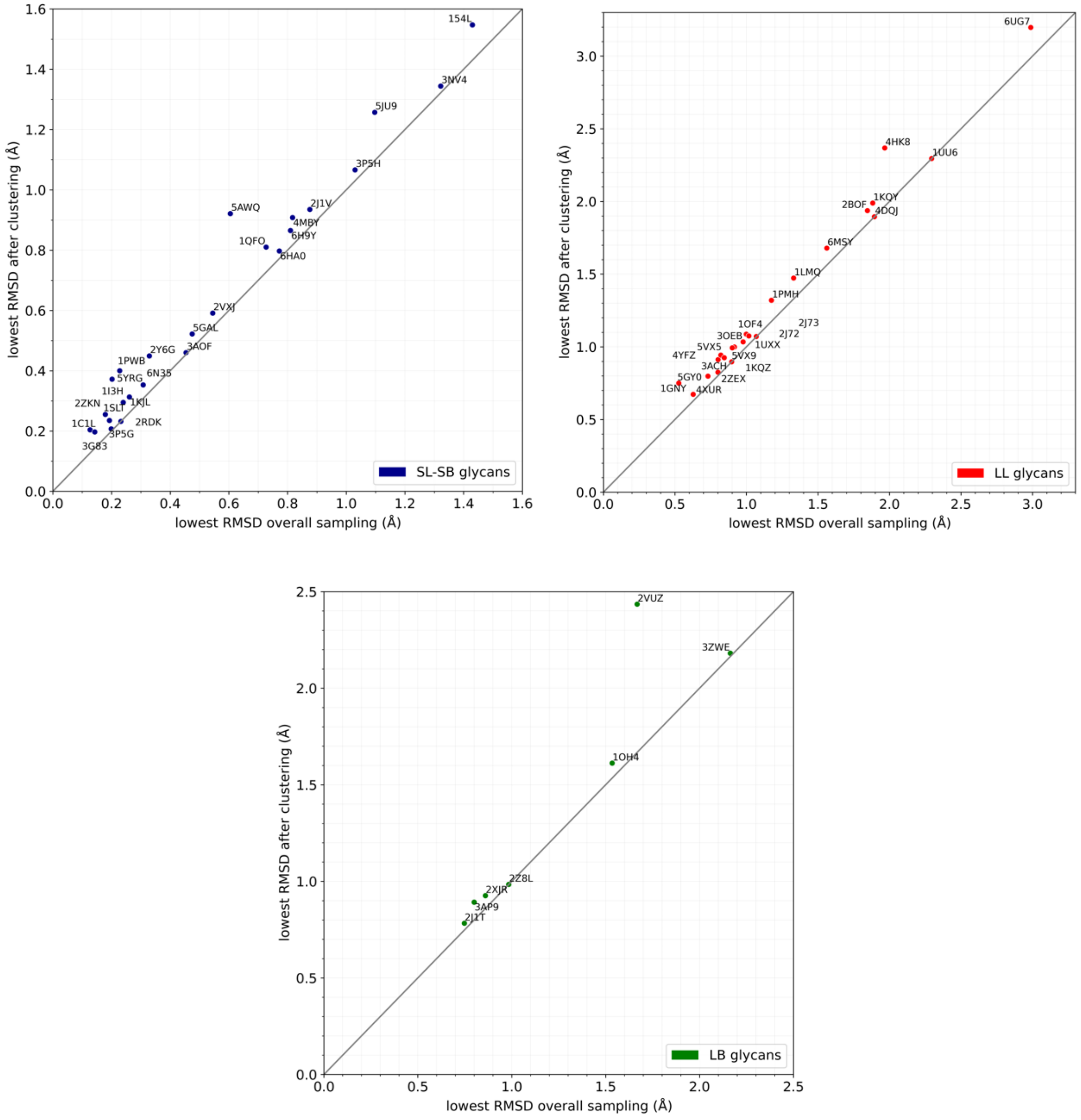

Figure S6

Superimposition of the centers of the 20 clusters (carbon atoms in light blue) obtained from the sf400-x16 sampling scenario and of the unbound conformation generated by GLYCAM-Web webserver (carbon atoms in yellow) to the bound conformations (carbon atoms in black) for the for the complexes 1OH4 (**LB**), 5VX5 (**LL**), and 1C1L (**SL**). Oxygen atoms are shown in red in all the structures, nitrogens in blue, hydrogens not shown.

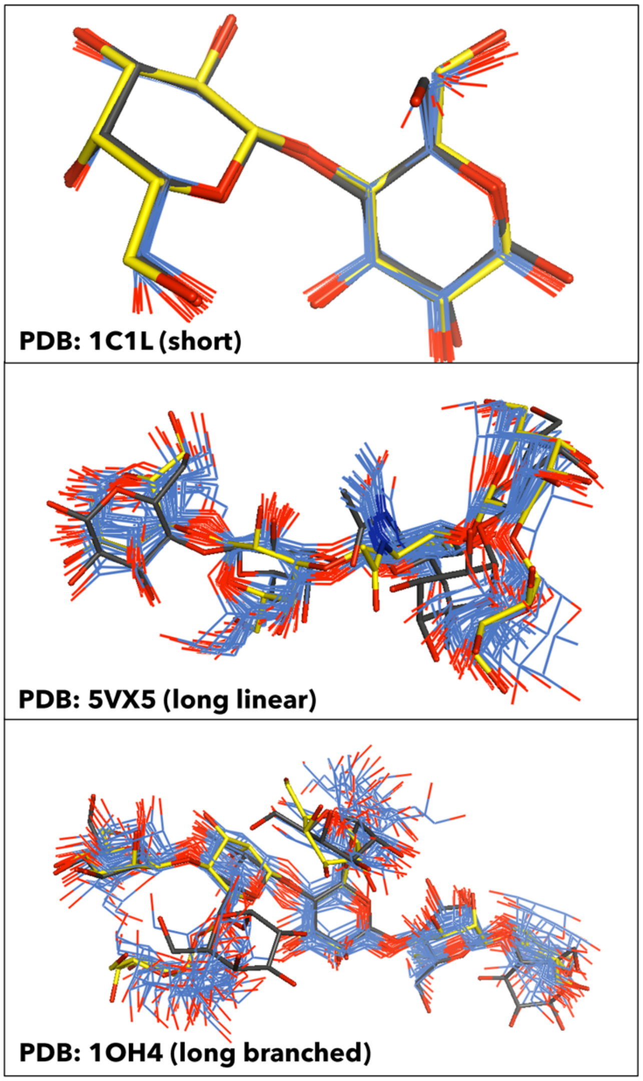

Figure S7

HADDOCK3 performance on the *unbound dataset*, using *vdW* scoring function and **tip-ap** AIRs. The success rates (SR), calculated on the top (T) 1, 5, 10, 50, 100, and 200 refined models (flexref stage), are compared between single conformations runs (left column) and ensemble runs (right column). SR are calculated for the entire dataset (first row), and separately for the three categories of complexes grouped by glycans size and connectivity: **SL-SB** (second row), **LL** (third row), and **LB** (fourth row).

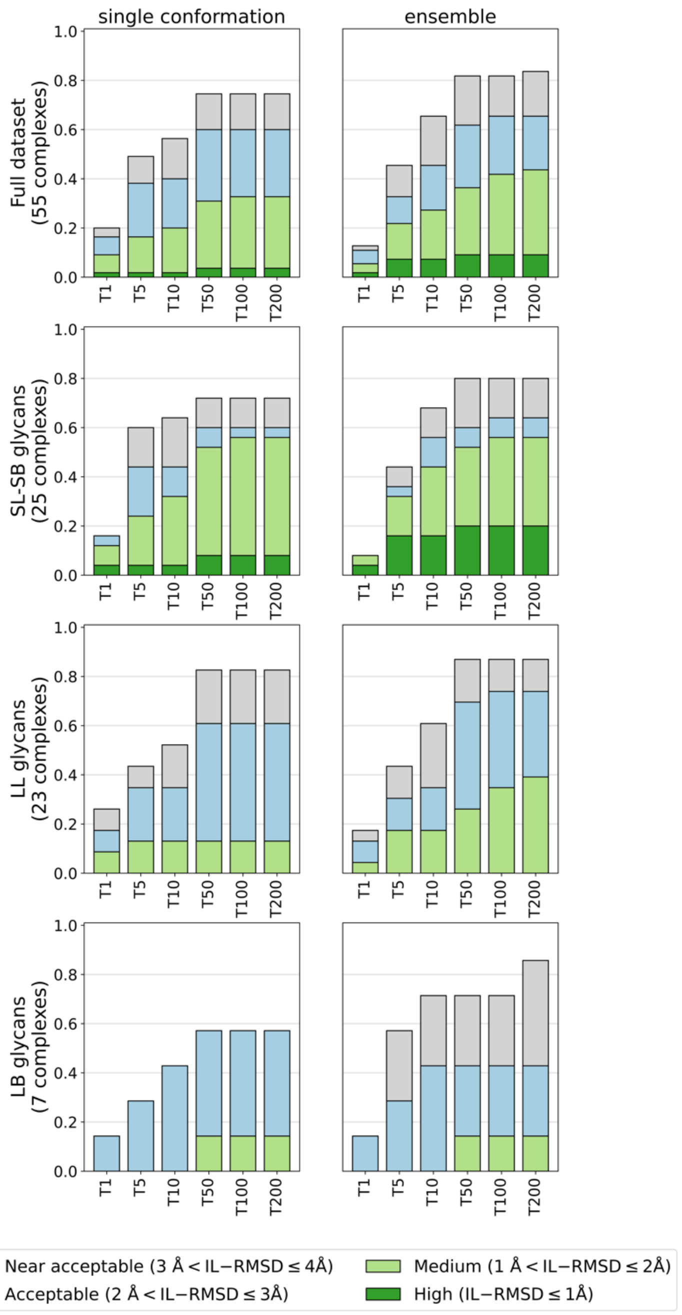

Figure S8

Comparison between ϕ and ψ dihedral angles of the 148 glycosidic linkages present in our "unbound" dataset. The values observed in the reference structure (red stars) are typically very similar to those calculated by the GLYCAM webserver models (blue circles), with the exception of a few notably difficult linkages. The values obtained upon refining the rigid-body poses by means of the HADDOCK flexible refinement ("HADDOCK flex", green dots) are generally localized around the GLYCAM values, thus indicating the chemical plausibility of the obtained models. In some interesting cases (such as the ϕ2-ψ2 pair in 2VUZ) some HADDOCK-refined poses possess torsion angles that are more similar to the reference value than the GLYCAM starting values. This highlights the capability of the HADDOCK refinement and force field used to induce meaningful conformational changes that can improve the quality of the docking poses.

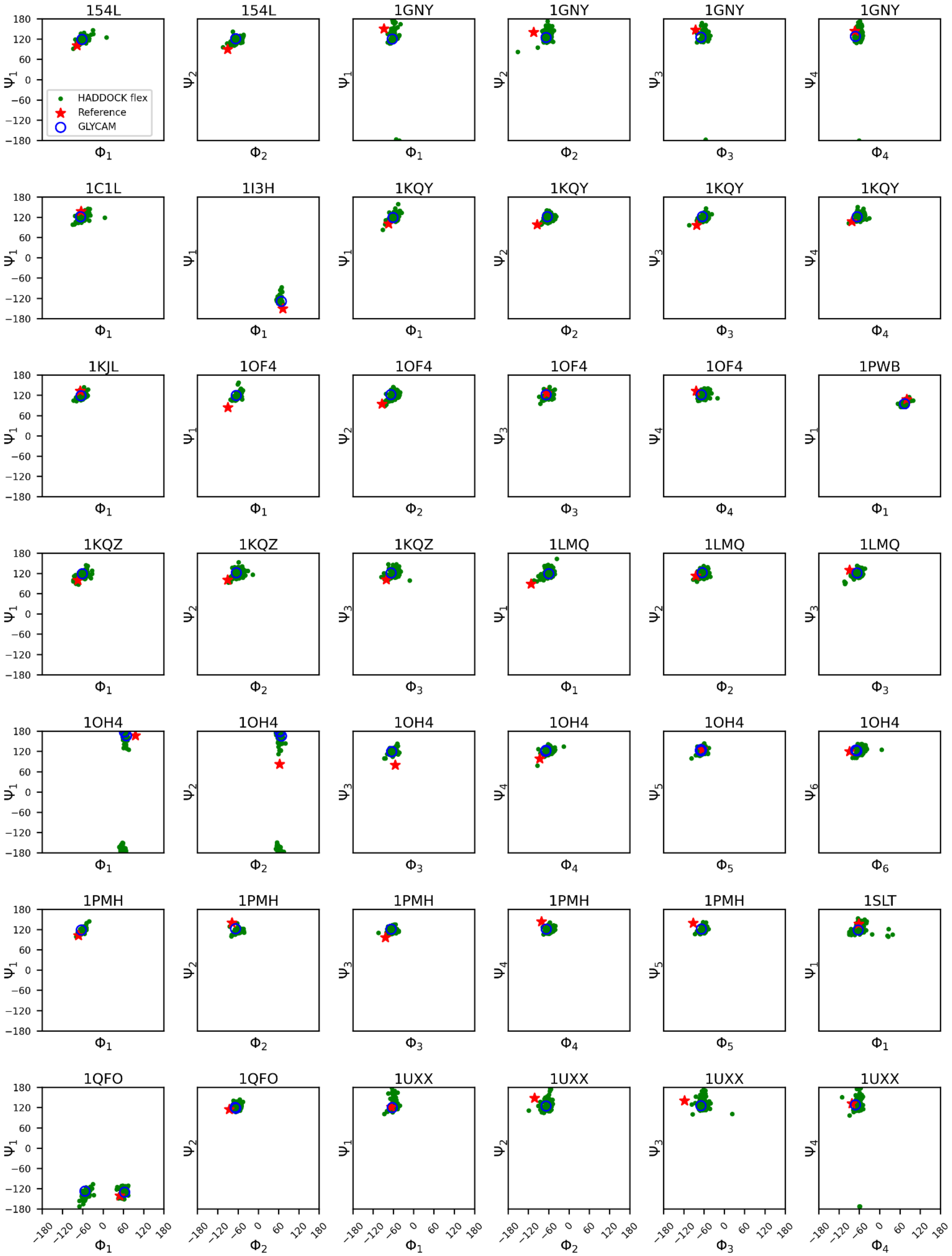
154L
154L
1GNY
1GNY
1GNY
1GNY
1C1L
1I3H
1KQY
1KQY
1KQY
1KQY
1KJL
1OF4
1OF4
1OF4
1OF4
1PWB
1KQZ
1KQZ
1KQZ
1LMQ
1LMQ
1LMQ
1OH4
1OH4
1OH4
1OH4
1OH4
1OH4
1PMH
1PMH
1PMH
1PMH
1PMH
1SLT
1QFO
1QFO
1UXX
1UXX
1UXX
1UXX
HADDOCK flex
Reference
GLYCAM
180
120
60
0
−60
−120
−180
−180
−120
−60
0
60
120
180
Φ_1
Φ_2
Φ_3
Φ_4
Φ_5
Φ_6
Ψ_1
Ψ_2
Ψ_3
Ψ_4
Ψ_5
Ψ_6

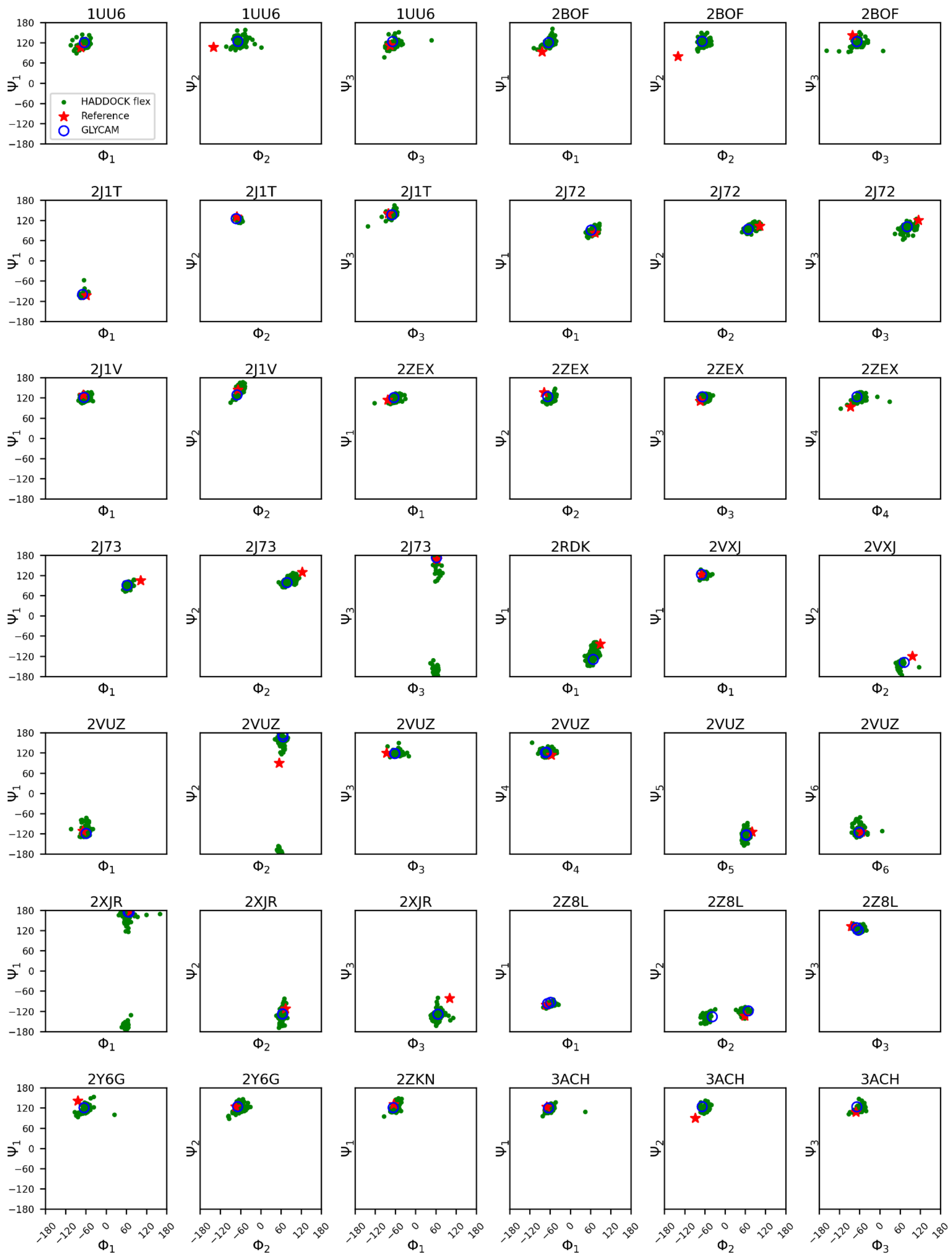
1UU6
2BOF
2J1T
2J72
2J1V
2ZEX
2J73
2RDK
2VXJ
2VUZ
2XJR
2Z8L
2Y6G
2ZKN
3ACH
HADDOCK flex
Reference
GLYCAM

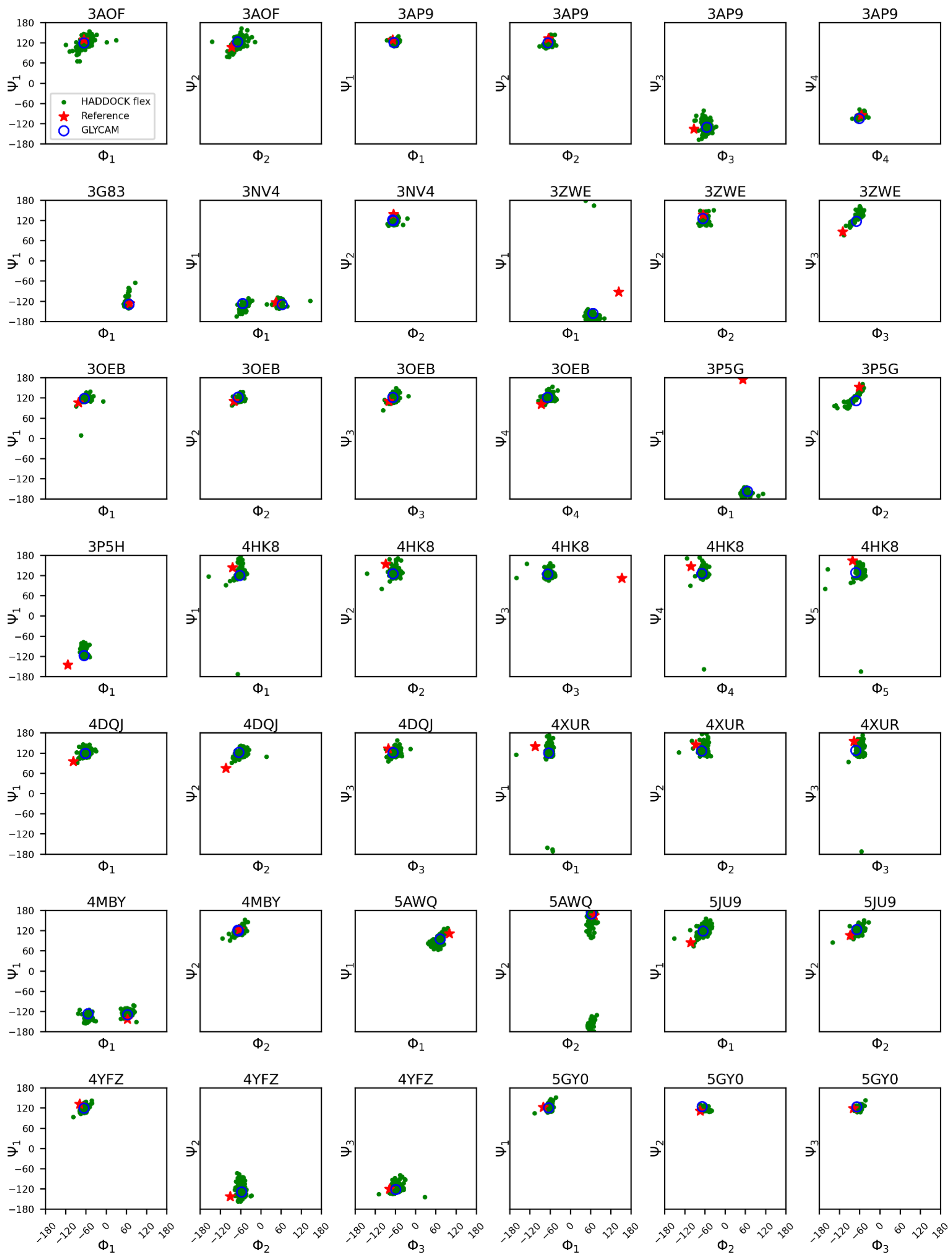
3AOF
3AOF
3AP9
3AP9
3AP9
3AP9
3G83
3NV4
3NV4
3ZWE
3ZWE
3ZWE
3OEB
3OEB
3OEB
3OEB
3P5G
3P5G
3P5H
4HK8
4HK8
4HK8
4HK8
4HK8
4DQJ
4DQJ
4DQJ
4XUR
4XUR
4XUR
4MBY
4MBY
5AWQ
5AWQ
5JU9
5JU9
4YFZ
4YFZ
4YFZ
5GY0
5GY0
5GY0
HADDOCK flex
Reference
GLYCAM
Φ1 Φ2 Φ3 Φ4 Φ5
Ψ1 Ψ2 Ψ3 Ψ4 Ψ5
180 120 60 0 −60 −120 −180

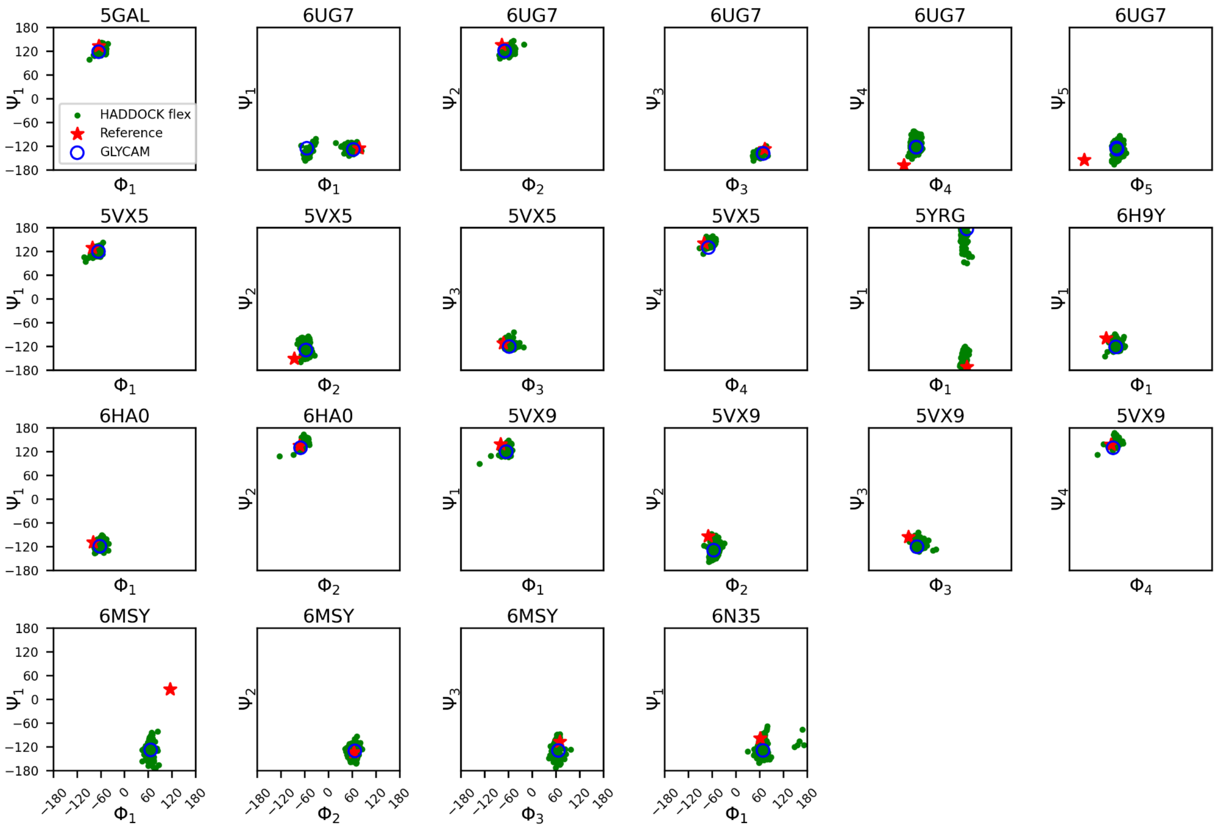

5GAL
6UG7
6UG7
6UG7
6UG7
6UG7
HADDOCK flex
Reference
GLYCAM
5VX5
5VX5
5VX5
5VX5
5YRG
6H9Y
6HA0
6HA0
5VX9
5VX9
5VX9
5VX9
6MSY
6MSY
6MSY
6N35
180
120
60
0
−60
−120
−180

Figure S9

Comparison between ϕ and ψ dihedral angles of the 148 glycosidic linkages present in our "unbound" dataset. Here the angles observed in the reference structure (red stars) and in the models generated by the GLYCAM webserver (blue circles) are compared to the values extracted after the short molecular dynamics refinement of the GLYCAM models ("HADDOCK MD", orange dots) described in the "Glycans conformational sampling" paragraph in the main text (see also Table S4). In general, such a short dynamics does not give rise to important changes in the values of these torsion angles, although in some challenging cases it is able to sample conformations that are closer to the reference structure. An example of this is represented by the $\phi2$-$\psi2$ pair in 1OH4.

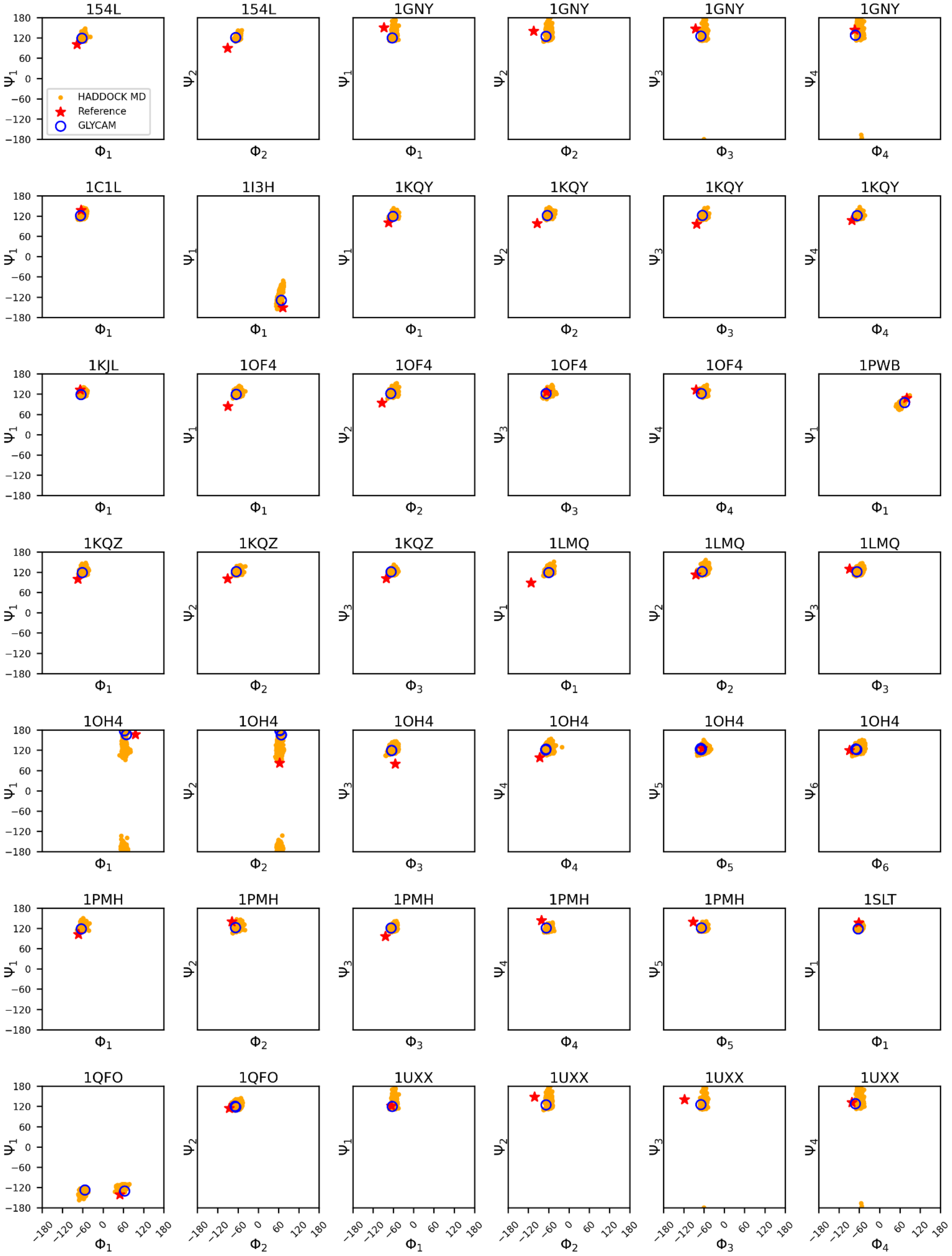
154L
154L
1GNY
1GNY
1GNY
1GNY
HADDOCK MD
Reference
GLYCAM
1C1L
1I3H
1KQY
1KQY
1KQY
1KQY
1KJL
1OF4
1OF4
1OF4
1OF4
1PWB
1KQZ
1KQZ
1KQZ
1LMQ
1LMQ
1LMQ
1OH4
1OH4
1OH4
1OH4
1OH4
1OH4
1PMH
1PMH
1PMH
1PMH
1PMH
1SLT
1QFO
1QFO
1UXX
1UXX
1UXX
1UXX
180
120
60
0
−60
−120
−180
Ψ_1
Ψ_2
Ψ_3
Ψ_4
Ψ_5
Ψ_6
Φ_1
Φ_2
Φ_3
Φ_4
Φ_5
Φ_6

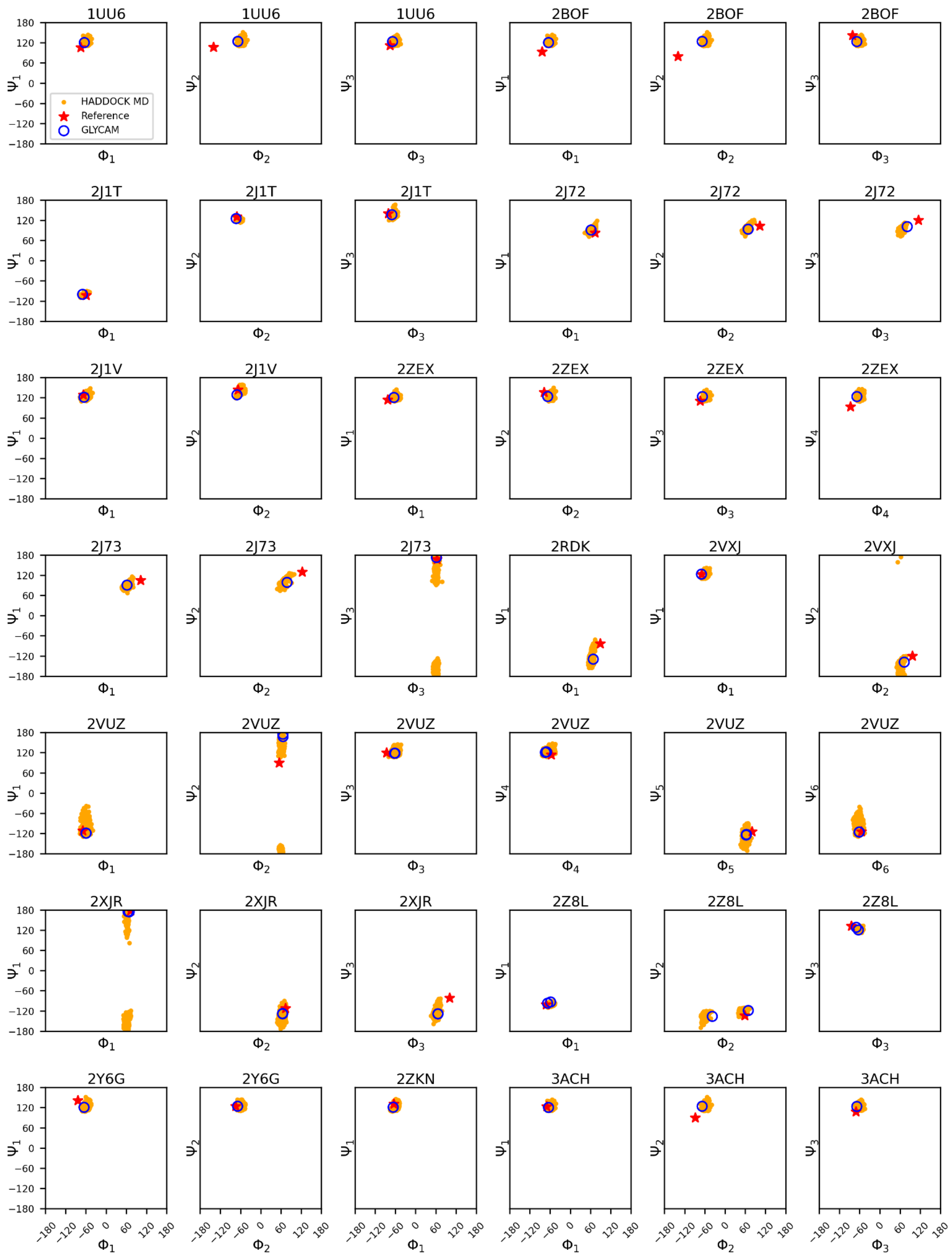

1UU6
1UU6
1UU6
2BOF
2BOF
2BOF
2J1T
2J1T
2J1T
2J72
2J72
2J72
2J1V
2J1V
2ZEX
2ZEX
2ZEX
2ZEX
2J73
2J73
2J73
2RDK
2VXJ
2VXJ
2VUZ
2VUZ
2VUZ
2VUZ
2VUZ
2VUZ
2XJR
2XJR
2XJR
2Z8L
2Z8L
2Z8L
2Y6G
2Y6G
2ZKN
3ACH
3ACH
3ACH
HADDOCK MD
Reference
GLYCAM

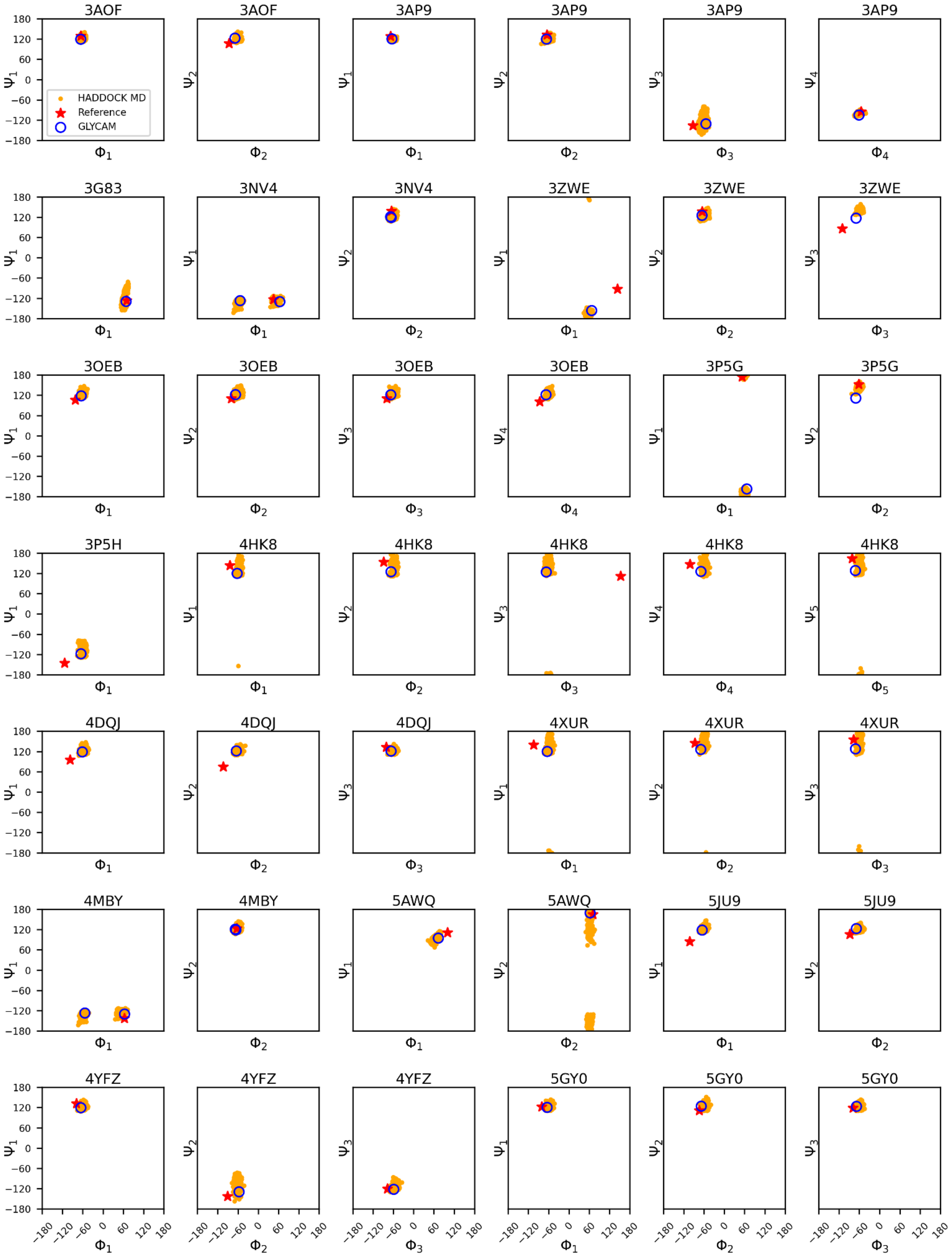
3AOF
3AOF
3AP9
3AP9
3AP9
3AP9
HADDOCK MD
Reference
GLYCAM
3G83
3NV4
3NV4
3ZWE
3ZWE
3ZWE
3OEB
3OEB
3OEB
3OEB
3P5G
3P5G
3P5H
4HK8
4HK8
4HK8
4HK8
4HK8
4DQJ
4DQJ
4DQJ
4XUR
4XUR
4XUR
4MBY
4MBY
5AWQ
5AWQ
5JU9
5JU9
4YFZ
4YFZ
4YFZ
5GY0
5GY0
5GY0

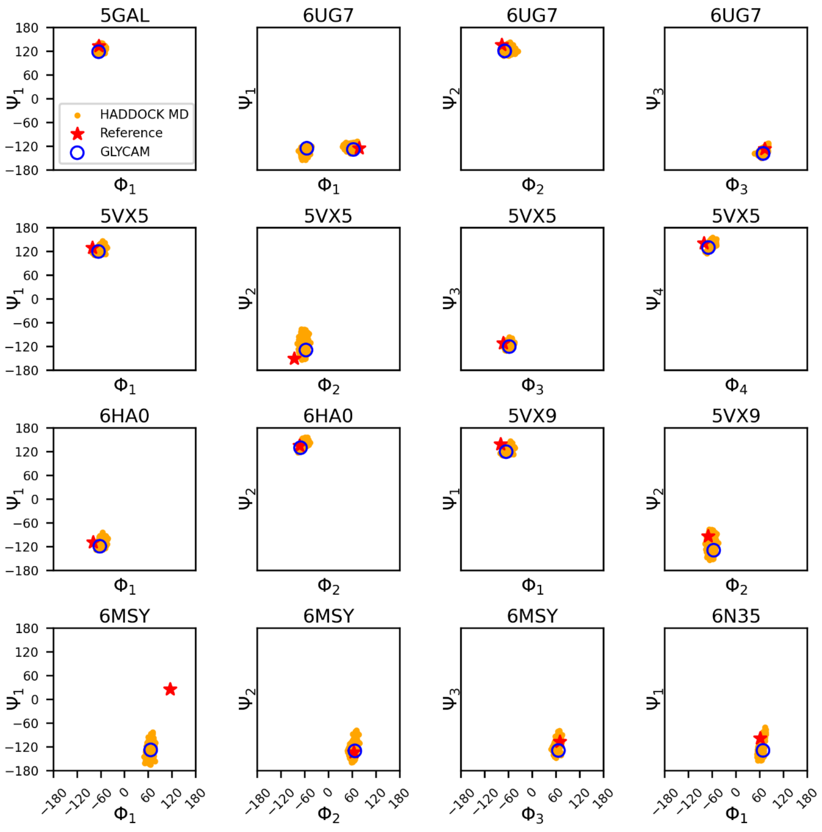

5GAL
6UG7
6UG7
6UG7
6UG7
6UG7
HADDOCK MD
Reference
GLYCAM
5VX5
5VX5
5VX5
5VX5
5YRG
6H9Y
6HA0
6HA0
5VX9
5VX9
5VX9
5VX9
6MSY
6MSY
6MSY
6N35

Supplementary References

(1) Woods Group. Complex Carbohydrate Research Center. GLYCAM Web, Http://Glycam.Org. University of Georgia, Athens, GA 2023. http://glycam.org.

(2) Kirschner, K. N.; Yongye, A. B.; Tschampel, S. M.; González-Outeiriño, J.; Daniels, C. R.; Foley, B. L.; Woods, R. J. GLYCAM06: A Generalizable Biomolecular Force Field. Carbohydrates. *J Comput Chem* 2008, *29* (4), 622–655. https://doi.org/10.1002/jcc.20820.

(3) Rodrigues, J. P. G. L. M.; Teixeira, J. M. C.; Trellet, M.; Bonvin, A. M. J. J. Pdb-Tools: A Swiss Army Knife for Molecular Structures. *F1000Res* 2018, *7*, 1961. https://doi.org/10.12688/f1000research.17456.1.

(4) Brünger, A. T.; Adams, P. D.; Clore, G. M.; DeLano, W. L.; Gros, P.; Grosse-Kunstleve, R. W.; Jiang, J. S.; Kuszewski, J.; Nilges, M.; Pannu, N. S.; Read, R. J.; Rice, L. M.; Simonson, T.; Warren, G. L. Crystallography & NMR System: A New Software Suite for Macromolecular Structure Determination. *Acta Crystallogr D Biol Crystallogr* 1998, *54* (5), 905–921. https://doi.org/10.1107/S0907444998003254.

(5) Janin, J.; Henrick, K.; Moult, J.; Eyck, L. Ten; Sternberg, M. J. E.; Vajda, S.; Vakser, I.; Wodak, S. J. CAPRI: A Critical Assessment of PRedicted Interactions. *Proteins: Structure, Function and Genetics* 2003, *52* (1), 2–9. https://doi.org/10.1002/prot.10381.

(6) Basu, S.; Wallner, B. DockQ: A Quality Measure for Protein-Protein Docking Models. *PLoS One* 2016, *11* (8), 1–9. https://doi.org/10.1371/journal.pone.0161879.

(7) Varki, A.; Cummings, R. D.; Aebi, M.; Packer, N. H.; Seeberger, P. H.; Esko, J. D.; Stanley, P.; Hart, G.; Darvill, A.; Kinoshita, T.; Prestegard, J. J.; Schnaar, R. L.; Freeze, H. H.; Marth, J. D.; Bertozzi, C. R.; Etzler, M. E.; Frank, M.; Vliegenthart, J. F.; Lütteke, T.; Perez, S.; Bolton, E.; Rudd, P.; Paulson, J.; Kanehisa, M.; Toukach, P.; Aoki-Kinoshita, K. F.; Dell, A.; Narimatsu, H.; York, W.; Taniguchi, N.; Kornfeld, S. Symbol Nomenclature for Graphical Representations of Glycans. *Glycobiology* 2015, *25* (12), 1323–1324. https://doi.org/10.1093/glycob/cwv091.

(8) Neelamegham, S.; Aoki-Kinoshita, K.; Bolton, E.; Frank, M.; Lisacek, F.; Lütteke, T.; O'Boyle, N.; Packer, N. H.; Stanley, P.; Toukach, P.; Varki, A.; Woods, R. J.; SNFG Discussion Group. Updates to the Symbol Nomenclature for Glycans Guidelines. *Glycobiology* 2019, *29* (9), 620–624. https://doi.org/10.1093/glycob/cwz045.

(9) Tsuchiya, S.; Aoki, N. P.; Shinmachi, D.; Matsubara, M.; Yamada, I.; Aoki-Kinoshita, K. F.; Narimatsu, H. Implementation of GlycanBuilder to Draw a Wide Variety of Ambiguous Glycans. *Carbohydr Res* 2017, *445*, 104–116. https://doi.org/https://doi.org/10.1016/j.carres.2017.04.015.

(10) Berman, H. M. The Protein Data Bank. *Nucleic Acids Res* 2000, *28* (1), 235–242. https://doi.org/10.1093/nar/28.1.235.
